# Histopathological Myocardial Changes and CPV-2 DNA Detection in Young Dogs: A Retrospective Study

**DOI:** 10.3390/vetsci13070643

**Published:** 2026-06-30

**Authors:** Adrian Stancu, Janos Degi, Iasmina Luca, Diana Maria Degi, Simona Marc, Sorin Aurelian Pașca, Sorin Octavian Voia, Adela Marcu

**Affiliations:** 1 Department of Pathological Anatomy, Faculty of Veterinary Medicine, University of Life Sciences “King Mihai I” from Timișoara, Calea Aradului 119, 300645 Timișoara, Romania; adrianstancu@usvt.ro (A.S.); iasmina.luca@usvt.ro (I.L.); diana.maria.degi@gmail.com (D.M.D.); simona.marc@usvt.ro (S.M.); 2 Department of Pathological Anatomy, Faculty of Veterinary Medicine, “Ion Ionescu de la Brad” Iași University of Life Sciences, Mihail Sadoveanu Alley, 700489 Iași, Romania; spasca@uaiasi.ro; 3 Department I–Animal Production Engineering, Faculty of Bioengineering of Animal Resources, University of Life Sciences “King Mihai I” from Timișoara, 300645 Timișoara, Romania; voia@animalsci-tm.ro (S.O.V.); adelamarcu@usvt.ro (A.M.)

**Keywords:** canine parvovirus, CPV-2, myocarditis, myocardial fibrosis, puppies, histopathology, PCR, cardiac pathology, veterinary pathology

## Abstract

Canine parvovirus type 2 (CPV-2) remains an important cause of morbidity and mortality in young dogs worldwide. Although the enteric form of the disease is well characterized, myocardial involvement associated with CPV-2 infection remains incompletely understood. The present retrospective study aimed to investigate myocardial lesions in young dogs and to evaluate their association with CPV-2 DNA detection using histopathological examination and PCR-based molecular analysis of formalin-fixed paraffin-embedded (FFPE) cardiac tissue. A total of 27 dogs aged between 3 and 11 months, submitted for diagnostic necropsy between 2019 and 2021, were included in the study. Histopathological evaluation was performed on myocardial tissue sections stained with hematoxylin and eosin, while conventional PCR targeting the VP1/VP2 region was used for the detection of CPV-2 DNA. Associations between PCR positivity and histopathological findings were assessed using Fisher’s exact test. Myocardial abnormalities were identified in all examined cases (27/27, 100%), with myocarditis consistently observed throughout the study population. Cardiomyocyte necrosis and myocardial fibrosis were identified in 9/27 (33.3%) and 14/27 (51.9%) cases, respectively. CPV-2 DNA was detected in 9/27 myocardial tissue samples (33.3%). Cardiomyocyte necrosis was significantly more frequent in CPV-2-positive dogs than in PCR-negative animals (77.8% vs. 11.1%; *p* ≈ 0.001). Similarly, myocardial fibrosis was identified more frequently in CPV-2-positive cases (100.0% vs. 27.8%; *p* ≈ 0.001). Myocarditis was present in all examined animals and therefore could not be evaluated statistically according to PCR status. The detection of CPV-2 DNA in myocardial tissues exhibiting histopathological lesions supports a possible association between CPV-2 infection and myocardial injury in young dogs. However, the retrospective design, limited sample size, and the use of conventional PCR do not allow conclusions regarding causality or direct viral involvement in lesion development. Further studies incorporating larger case series and complementary diagnostic techniques are needed to clarify the pathogenesis and clinical significance of CPV-2-associated myocardial lesions.

## Simple Summary

Canine parvovirus type 2 (CPV-2) is widely recognized as a major cause of severe enteritis in puppies; however, cardiac involvement remains insufficiently investigated. This retrospective study evaluated myocardial lesions in 27 young dogs submitted for diagnostic necropsy in Western Romania using histopathology and PCR-based molecular detection from formalin-fixed paraffin-embedded cardiac tissue. CPV-2 DNA was identified in one-third of the examined cases and was significantly associated with myocardial necrosis, inflammation, and fibrosis. The findings suggest that myocardial injury associated with CPV-2 infection may be more common than clinically recognized, particularly in puppies with sudden death or concurrent enteric disease. The study highlights the diagnostic value of combining histopathological examination with molecular testing in postmortem investigations of suspected canine parvoviral infection.

## 1. Introduction

Canine parvovirus type 2 (CPV-2) remains one of the most important viral pathogens affecting domestic dogs worldwide, particularly puppies younger than six months of age. The infection is primarily associated with acute hemorrhagic enteritis, severe dehydration, leukopenia, and high mortality rates in unvaccinated animals despite the widespread availability of vaccines [1,2,3,4]. Since its emergence in the late 1970s, CPV-2 has remained an important canine pathogen worldwide and continues to be associated with substantial morbidity and mortality despite widespread vaccination [5,6,7].

Although the gastrointestinal form of the disease is well recognized, cardiac involvement associated with CPV-2 infection is considerably less frequently reported. Parvoviral myocarditis is typically described in neonatal or very young puppies and is characterized by myocardial necrosis, lymphohistiocytic inflammation, and variable degrees of myocardial fibrosis [1,8]. The susceptibility of young puppies is believed to be associated with the high mitotic activity of cardiomyocytes during early postnatal development, which provides favorable conditions for parvoviral replication [9,10].

In recent years, several studies have suggested that myocardial involvement in CPV infection may be underrecognized, particularly in puppies that die suddenly or present predominantly enteric clinical signs [1,11]. However, the prevalence and histopathological spectrum of CPV-associated cardiac lesions remain incompletely characterized, especially in naturally infected dogs. Furthermore, molecular detection of CPV DNA in formalin-fixed paraffin-embedded (FFPE) myocardial tissue has only been rarely investigated in retrospective pathological studies.

CPV-2 has been previously associated with myocarditis and myocardial fibrosis, particularly in young dogs, although the mechanisms and direct viral involvement remain incompletely clarified.

The association between CPV-2 infection and myocardial injury, particularly in neonatal and very young puppies, has been previously documented and is not considered a novel pathological entity. However, retrospective documentation of CPV-2-associated myocardial lesions from archived diagnostic material remains limited in some geographic regions, including Romania. Therefore, the present study aimed to describe the histopathological findings and PCR-based detection of CPV-2 DNA in FFPE myocardial tissue from young dogs submitted for diagnostic necropsy in Western Romania.

## 2. Materials and Methods

### 2.1. Case Selection and Study Design

This retrospective observational study was conducted using archived necropsy material collected at the Laboratory of Pathological Anatomy, University Veterinary Clinics, Faculty of Veterinary Medicine, Timișoara, Romania. The study included 27 puppies submitted for postmortem examination between May 2019 and December 2021 with a clinical suspicion of canine parvoviral infection or sudden unexplained death.

Clinical information was retrieved retrospectively from submission forms and available medical records. When clinical signs were not documented, cases were recorded as having an unavailable or incomplete clinical history rather than being classified as asymptomatic.

Cases were selected based on the availability of formalin-fixed paraffin-embedded (FFPE) cardiac tissue, complete necropsy records, and adequate histopathological preservation of myocardial samples. Puppies younger than one year of age were eligible for inclusion irrespective of breed or sex. Cases presenting severe autolysis or insufficient myocardial tissue were excluded from the study.

Clinical history, age, sex, breed status, and gross pathological findings were retrieved from necropsy records whenever available.

### 2.2. Gross Pathology and Histopathology

Complete necropsies were performed according to standard veterinary pathological protocols. Cardiac tissue samples, including sections from the left and right ventricular free walls and interventricular septum, were routinely collected during postmortem examination.

Tissues were fixed in 10% neutral-buffered formalin, processed routinely, embedded in paraffin wax, sectioned at 4–5 μm thickness, and stained with hematoxylin and eosin (H&E).

Lesion severity was assessed descriptively and was not used as a formal quantitative variable for statistical analysis.

Histopathological evaluation was performed independently by a veterinary pathologist blinded to the PCR results. Myocardial lesions were assessed for the presence of cardiomyocyte degeneration and necrosis, inflammatory infiltrates, interstitial or replacement fibrosis, and vascular alterations. Inflammatory infiltrates were characterized according to their predominant cellular composition.

### 2.3. DNA Extraction from FFPE Cardiac Tissue

Genomic DNA was extracted from FFPE myocardial tissue using the QIAamp DNA FFPE Tissue Kit (QIAGEN, Hilden, Germany), according to the manufacturer’s instructions.

Briefly, 10 μm paraffin sections were deparaffinized, followed by tissue lysis and DNA purification. DNA concentration and purity were evaluated spectrophotometrically prior to PCR analysis.

To assess DNA integrity and suitability for amplification, a housekeeping gene (RPS19) was amplified in all samples before CPV-specific PCR testing.

### 2.4. PCR Detection of Canine Parvovirus Type 2

Conventional PCR was used to detect CPV-2 DNA in FFPE myocardial samples. This method confirms the presence of viral genetic material but does not demonstrate active viral replication, intralesional viral localization, or direct virus-induced myocardial damage.

Amplification was performed using previously published primers targeting the VP1/VP2 region of the CPV genome: forward primer CPV-940F (5′-CGTGGTGTAACTCAAATGGGAAA-3′) and reverse primer PV-1617R (5′-GGATTCCAAGTATGAGAGGCTCTT-3′). PCR products were analyzed by agarose gel electrophoresis, and positive samples yielded an expected amplicon of 678 bp, depending on the reference genome used for alignment.

Sequencing was not performed in the present study. Therefore, the genetic identity of the detected CPV-2 strains, their relationship to circulating field strains, and their distinction from vaccine-derived strains could not be assessed.

Positive and negative controls were included in each PCR run. The positive control consisted of intestinal tissue previously confirmed as CPV-2 positive by viral isolation and PCR, while nuclease-free water was used as the no-template negative control.

### 2.5. Statistical Analysis

Statistical analyses were performed using Fisher’s exact test to compare the frequency of histopathological lesions between CPV-2-positive and CPV-2-negative groups.

Categorical variables were expressed as absolute numbers and percentages. A *p*-value < 0.05 was considered statistically significant.

Given the relatively small sample size, statistical findings were interpreted cautiously and considered exploratory.

All diagnostic procedures and sample submissions were performed with owner consent obtained at the time of clinical admission or necropsy submission and institutional collaboration agreements concluded with the Faculty of Veterinary Medicine, Timisoara (328/11 September 2024), in accordance with the provisions of Romanian Law No. 160/1998 regarding the organization and practice of the veterinary profession, as well as the Decision of the National Council of the Romanian College of Veterinarians No. 34/1 December 2012 concerning informed consent for veterinary medical procedures.

### 2.6. Use of Artificial Intelligence-Assisted Language Editing

During manuscript preparation, ChatGPT-5.5 (OpenAI, San Francisco, CA, USA) was used exclusively to improve English language quality, grammar, readability, and academic phrasing. The tool was not used for study design, data collection, data analysis, statistical evaluation, histopathological interpretation, molecular diagnostics, or formulation of scientific conclusions. All generated text was critically reviewed, edited, and verified by the authors, who assume full responsibility for the final content of the manuscript.

## 3. Results

### 3.1. Demographic and Clinical Characteristics

A total of 27 puppies met the inclusion criteria and were included in the study (Table S1—Supplementary File). The demographic and clinical characteristics of the investigated cases are summarized in Table 1.

The age of the animals ranged from 3 to 11 months. Twelve dogs were 3 months old, five were 6 months old, two were 7 months old, six were 10 months old, and two were 11 months old. Fourteen dogs were male and thirteen were female. Mixed-breed dogs represented 9/27 cases (33.3%), whereas purebred dogs accounted for 18/27 cases (66.7%).

Based on the available clinical records, 22/27 puppies had a documented history of diarrhea, while the remaining cases were submitted for necropsy following sudden death, with no previous clinical signs recorded or with incomplete clinical information. Histopathological myocardial lesions were identified in all 27 examined cases (100%). CPV-2 DNA was detected by PCR in myocardial tissue samples from 9 puppies (33.3%), while the remaining 18 cases (66.7%) tested negative.

### 3.2. PCR Detection and Molecular Characterization of CPV-2

Conventional PCR targeting the VP1/VP2 region detected CPV-2 DNA in 9 of the 27 myocardial tissue samples (33.3%). Positive samples produced amplicons of the expected size on agarose gel electrophoresis, whereas no amplification was observed in the negative controls. Since sequencing was not performed, the molecular findings were limited to PCR-based detection of CPV-2 DNA. No amplification was detected in negative controls included in the PCR assays.

CPV-2 DNA detection was recorded in myocardial tissues presenting histopathological lesions.

Representative PCR amplification results are presented in Figures S1–S3.

### 3.3. Gross Pathological Findings

At necropsy, several puppies showed cardiomegaly with multifocal pale areas affecting the ventricular myocardium. Pale discoloration was observed predominantly within the free walls of the left and right ventricles.

Small amounts of serosanguineous fluid were present within the thoracic and abdominal cavities in several cases. Additional gross findings included pulmonary congestion and hepatic congestion of variable severity.

In puppies with concurrent enteric disease, intestinal lesions compatible with parvoviral enteritis were also observed.

### 3.4. Histopathological Findings

Histopathological examination revealed myocardial abnormalities in all 27 investigated puppies, with myocarditis being consistently observed across all cases. Additional lesions, including cardiomyocyte necrosis and myocardial fibrosis, were identified in 9/27 (33.3%) and 14/27 (51.9%) cases, respectively, as detailed in Supplementary Table S3.

The most frequently observed lesions included cardiomyocyte degeneration and necrosis, multifocal inflammatory infiltrates, and interstitial or replacement fibrosis. Inflammatory infiltrates were composed predominantly of lymphocytes, macrophages, plasma cells, and occasional neutrophils.

A subset of puppies showed concurrent cardiomyocyte necrosis, inflammation, and fibrosis, whereas 14/27 cases showed a combination of two or more cardiac lesions, as detailed in Supplementary Table S3.

No definitive viral intranuclear inclusion bodies were consistently identified within cardiomyocytes.

Fibrotic lesions were characterized by replacement of myocardial fibers with eosinophilic collagenous connective tissue associated with disruption of normal myocardial architecture (Figure 1).

Epicardial alterations, including mesothelial disruption and superficial inflammatory exudate, were observed in several cases and were consistent with mild fibrinous epicarditis (Figure 2).

Additionally, 5 cases (18.5%) showed predominantly necrotic myocardial lesions, whereas 3 cases (11.1%) were characterized mainly by myocardial fibrosis (Tables S2 and S3). Representative microphotographs of these lesions are provided in Figure 1 and Figure 2. No definitive viral intranuclear inclusion bodies were consistently identified within cardiomyocytes.

Histopathological examination revealed myocardial abnormalities in all 27 examined cases, primarily consisting of myocarditis. Additional lesions, including cardiomyocyte necrosis and myocardial fibrosis, were observed in 9/27 (33.3%) and 14/27 (51.9%) cases, respectively, as detailed in Supplementary Table S3.

### 3.5. Association Between CPV-2 Detection and Myocardial Lesions

Cardiomyocyte necrosis and myocardial fibrosis were more frequently recorded in CPV-2-positive myocardial samples than in CPV-2-negative samples.

Myocarditis was observed in all examined cases, irrespective of PCR status, and therefore was not suitable for comparative statistical analysis.

Cardiomyocyte necrosis was significantly more frequent in CPV-2-positive puppies than in CPV-2-negative cases (77.8% vs. 11.1%; *p* ≈ 0.001).

Myocardial fibrosis was also significantly associated with CPV-2 positivity, being identified in 100% of positive cases compared with 27.8% of negative cases (*p* ≈ 0.001) (Table 2).

Puppies positive for CPV-2 DNA were more likely to present multiple concurrent myocardial lesions than PCR-negative animals.

**Table 2:** Association Between CPV-2 Detection and Myocardial Histopathological Lesions.

Histopathological Lesion	CPV-2 Positive (n = 9)	CPV-2 Negative (n = 18)	Total (n = 27)	Fisher’s Exact Test *
Cardiomyocyte necrosis	7 (77.8%)	2 (11.1%)	9 (33.3%)	*p* ≈ 0.001
Myocardial fibrosis	9 (100.0%)	5 (27.8%)	14 (51.9%)	*p* ≈ 0.001
Myocarditis	9 (100.0%)	18 (100.0%)	27 (100.0%)	Not applicable
Combination of cardiac lesions	9 (100.0%)	5 (27.8%)	14 (51.9%)	*p* ≈ 0.005

* Statistically significant at *p* < 0.05.

## 4. Discussion

The findings of the present study should be interpreted in the context of previous evidence showing that CPV-2 can cause myocarditis and myocardial fibrosis in young puppies. Therefore, the present data do not establish a new disease association or provide mechanistic insight into CPV-2 pathogenesis. Rather, they provide retrospective regional documentation of CPV-2 DNA detection in myocardial tissue and its association with histopathological cardiac lesions in a group of puppies from Romania. The study should therefore be regarded as a regional retrospective diagnostic case series rather than as evidence of a novel pathogenic association.

The myocardial lesions observed in the present study, including degeneration, necrosis, inflammatory infiltrates, and fibrosis, are not specific to CPV-2 infection and may result from a variety of systemic or infectious conditions. Severe parvoviral enteritis can be associated with dehydration, hypovolemia, endotoxemia, septicemia, electrolyte imbalances, and tissue hypoxia, all of which may contribute to secondary myocardial injury. Therefore, the histopathological findings should be interpreted within the broader clinical and pathological context.

Because broader pathogen screening, immunohistochemistry, in situ hybridization, and other techniques capable of demonstrating viral localization within myocardial lesions were not included in the present study, alternative infectious and non-infectious causes of myocardial injury cannot be completely excluded.

The classical mechanism of CPV-associated myocarditis has been mainly described in neonatal or very young puppies, in which viral replication may be favored by the mitotic activity of cardiomyocytes. However, this mechanism cannot be directly extrapolated to older puppies included in the present study, such as those aged 6, 7, 10, or 11 months. In these animals, the detection of CPV-2 DNA in myocardial tissue should be interpreted cautiously and may reflect systemic viral dissemination, persistence of viral DNA, secondary myocardial injury, or an association with concurrent pathological processes rather than direct infection of actively dividing cardiomyocytes.

Although canine parvoviral enteritis remains the most widely recognized manifestation of CPV infection, cardiac involvement continues to be considered relatively uncommon in routine clinical practice. Historically, parvoviral myocarditis has been described predominantly in neonatal or very young puppies infected during the early postnatal period, when cardiomyocytes still retain mitotic activity necessary for parvoviral replication [1,2]. The dogs included in the present study ranged in age from 3 to 11 months. Therefore, the classical pathogenesis described in neonatal puppies cannot be directly extrapolated to all cases included in this study, and the detection of CPV-2 DNA should be interpreted cautiously [1,9].

The histopathological lesions observed in this study, including myocardial necrosis, mononuclear inflammation, and interstitial fibrosis, are in agreement with previously described patterns of CPV-associated myocarditis [1,11]. In several cases, fibrosis coexisted with active inflammatory lesions, suggesting that myocardial injury may persist beyond the acute phase of infection and contribute to chronic myocardial remodeling. Similar findings have been reported in both experimental and naturally occurring CPV infections, where replacement fibrosis has been associated with progressive myocardial dysfunction and delayed cardiac failure [1,2].

An important finding of the present study was the statistically significant association between CPV-2 detection and the severity of myocardial lesions. Puppies positive for CPV-2 DNA showed a higher frequency of cardiomyocyte necrosis and myocardial fibrosis compared with PCR-negative animals. Because myocarditis was identified in all examined puppies, no statistical comparison according to PCR status was possible for this lesion. Nevertheless, myocardial lesions were also identified in some PCR-negative cases, indicating that additional infectious or noninfectious factors may also play a role in the development of cardiac injury.

Potential contributing factors may include systemic hypoxia, septicemia, metabolic disturbances, or coinfections with other cardiotropic pathogens. Because additional virological or bacteriological investigations were not performed systematically in all cases, the contribution of concurrent infectious agents cannot be excluded. Future studies incorporating immunohistochemistry, in situ hybridization, or broader pathogen screening approaches may help further clarify the etiopathogenesis of myocardial lesions in puppies with suspected viral myocarditis.

Because sequencing was not performed, the present study cannot provide information regarding the genetic characteristics of the detected viral strains. Therefore, the molecular findings are limited to the detection of CPV-2 DNA in myocardial tissue. This observation is consistent with recent reports describing the increasing prevalence of CPV-2c in several geographic regions worldwide [12,13,14,15,16]. Although the pathogenic significance of different CPV variants in myocardial disease remains incompletely understood, continued molecular surveillance remains important for monitoring viral evolution and vaccine effectiveness [17,18,19,20,21,22,23,24,25,26,27].

The use of FFPE myocardial tissue represented both a practical advantage and a methodological limitation of the study. Retrospective analysis of archived pathological material allowed evaluation of naturally occurring cases over multiple years; however, formalin fixation may reduce nucleic acid quality and potentially decrease PCR sensitivity. Consequently, false-negative results cannot be completely excluded, particularly in cases with low viral DNA concentrations or prolonged tissue fixation.

Several additional limitations should be acknowledged. First, the relatively small sample size limited the statistical power of the analyses and prevented more advanced multivariable modeling. Second, detailed vaccination histories were unavailable for some puppies, precluding assessment of vaccine status or potential vaccine failure. Third, molecular investigation was limited to conventional PCR detection, and no sequencing or variant-level characterization was performed.

Because this was a retrospective study, clinical information was dependent on the completeness of submission forms and medical records. Therefore, the absence of recorded clinical signs should not be interpreted as confirmation that the animals were clinically asymptomatic.

Although CPV-2 DNA was detected in myocardial tissues with histopathological lesions, the present study cannot establish a definitive causal relationship between viral infection and myocardial injury. Conventional PCR performed on FFPE tissue confirms the presence of viral DNA but does not indicate active viral replication or localization of the virus within specific myocardial lesions. Therefore, the findings should be interpreted as evidence of an association rather than proof of direct CPV-2-induced myocardial damage.

Because PCR amplicons were not sequenced, the present study cannot assign the detected CPV-2 strains to a specific antigenic variant. Therefore, no conclusions can be drawn regarding the circulation of CPV-2a, CPV-2b, or CPV-2c in the investigated cases. Future studies including sequencing, GenBank deposition, and phylogenetic analysis are needed to characterize the CPV-2 variants involved in myocardial tissue infection and in situ hybridization, immunohistochemistry, quantitative PCR, viral RNA detection, or other methods demonstrating viral localization and replication within myocardial cells would be necessary to clarify the pathogenic role of CPV-2 in myocardial injury.

A further limitation of the study is that PCR-positive amplicons were not sequenced. Consequently, the study confirms the presence of CPV-2 DNA in myocardial tissue but does not provide variant-level molecular characterization.

Given the limited sample size and the relatively small number of PCR-positive cases, the statistical analyses should be regarded as exploratory. Consequently, the observed associations should be interpreted cautiously and validated in larger studies.

Despite these limitations, the study highlights the importance of considering myocardial involvement in puppies with suspected CPV infection, particularly in cases of sudden death or atypical clinical presentation. The combination of histopathological examination and molecular detection from myocardial tissue may improve postmortem diagnostic accuracy and contribute to a better understanding of the cardiac manifestations associated with CPV infection in young dogs.

## 5. Conclusions

The present study documents CPV-2 DNA detection in FFPE myocardial tissue from young dogs in Romania and describes associated myocardial lesions, including necrosis, inflammation, and fibrosis. These findings are consistent with previously recognized patterns of CPV-2-associated myocardial injury. The study should therefore be regarded as a regional retrospective diagnostic case series rather than as evidence of a novel pathogenic association.

The findings support a possible association between CPV-2 DNA detection and myocardial lesions in young dogs. However, further studies are required to confirm whether CPV-2 plays a direct causal role in the development of these cardiac changes.

Although the retrospective design and the use of FFPE tissue impose certain methodological limitations, the combined use of histopathology and PCR analysis proved valuable for identifying cardiac involvement associated with CPV-2 infection.

Further investigations including larger case series, broader pathogen screening, immunohistochemistry, and complete molecular characterization of circulating viral strains are needed to better define the pathogenesis and clinical significance of CPV-associated myocardial lesions in dogs.

## Figures and Tables

**Figure 1:**
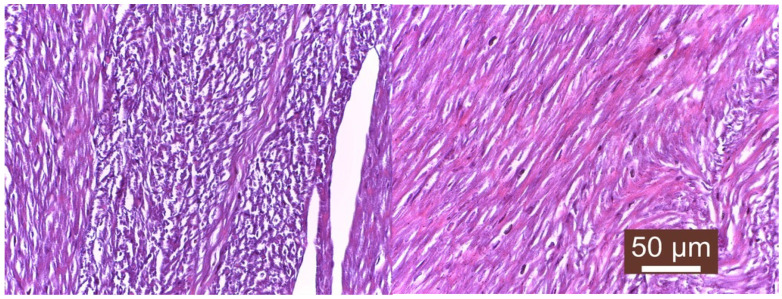
Interstitial and replacement myocardial fibrosis characterized by collagen deposition and disruption of normal myocardial architecture (H&E stain, scale bar = 50 μm).

**Figure 2:**
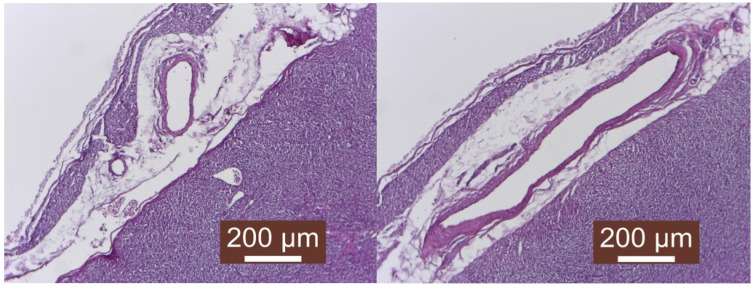
Desquamation of the epicardial mesothelium and associated inflammatory exudate overlying cardiac tissue (H&E stain, scale bar = 200 µm).

**Table 1:** Demographic and Clinical Characteristics of the Studied Puppies.

Variable	N (%)
Total cases	27 (100%)
Age 3 months	12 (44.4%)
Age 6 months	5 (18.5%)
Age 7 months	2 (7.4%)
Age 10 months	6 (22.2%)
Age 11 months	2 (7.4%)
Male	14 (51.9%)
Female	13 (48.1%)
Purebred	18 (66.7%)
Mixed breed	9 (33.3%)
CPV-2 positive (PCR)	9 (33.3%)
CPV-2 negative (PCR)	18 (66.7%)
Myocarditis	27 (100%)
Cardiomyocyte necrosis	9 (33.3%)
Myocardial fibrosis	14 (51.9%)
Clinical history of diarrhea	22 (81.5%)

## Data Availability

The original contributions presented in this study are included in the article/Supplementary Materials. Further inquiries can be directed to the corresponding author.

## Supplementary Materials

The following supporting information can be downloaded at: https://www.mdpi.com/article/10.3390/vetsci13070643/s1, Table S1. Epidemiological and clinical data of the investigated dogs; Table S2: Pathological findings and PCR-based detection of CPV-2 DNA in myocardial tissue samples from young dogs; Table S3: Distribution of myocardial histopathological lesions according to CPV-2 DNA detection status; Figure S1: PCR detection of CPV-2 DNA in myocardial tissue samples (samples S1–S12); Figure S2: PCR detection of CPV-2 DNA in myocardial tissue samples (samples S13–S24); Figure S3: PCR detection of CPV-2 DNA in myocardial tissue samples (samples S25–S27).
