# Supplementary material for: Histopathological Myocardial Changes and CPV-2 DNA Detection in Young Dogs: A Retrospective Study
Source: Vet Sci. 2026 Jun 30;13(7):643. doi: 10.3390/vetsci13070643 (PMC13417702; doi:10.3390/vetsci13070643)
Supplement: Supplementary file 1 [file vetsci-13-00643-s001.zip › vetsci-4345293-supplementary.pdf]

Table S1. Epidemiological and clinical data of the investigated dogs

Case ID	Breed	Sex	Age	Vaccination status	Clinical history
RO01DJ	German Shepherd	M	3 months	Complete vaccination	Diarrhea prior to death
RO02DJ	Labrador Retriever	F	6 months	Incomplete vaccination	Diarrhea prior to death
RO03DJ	Boxer	F	3 months	Complete vaccination	Diarrhea prior to death
RO04DJ	German Shepherd	M	10 months	Incomplete vaccination	Sudden death; previous clinical signs not documented
RO05DJ	French Bulldog	F	3 months	Unvaccinated	Diarrhea prior to death
RO06DJ	Golden Retriever	M	6 months	Unvaccinated	Diarrhea prior to death
RO07DJ	Mixed breed	F	10 months	Unvaccinated	Diarrhea prior to death
RO08DJ	Labrador Retriever	F	3 months	Incomplete vaccination	Diarrhea prior to death
RO09DJ	Siberian Husky	M	7 months	Unvaccinated	Sudden death; previous clinical signs not documented
RO10DJ	Mixed breed	M	11 months	Complete vaccination	Diarrhea prior to death
RO11DJ	Mixed breed	F	3 months	Incomplete vaccination	Diarrhea prior to death
RO12DJ	Mixed breed	M	10 months	Unvaccinated	Diarrhea prior to death
RO13DJ	Siberian Husky	F	3 months	Unvaccinated	Sudden death; previous clinical signs not documented
RO14DJ	French Bulldog	M	7 months	Complete vaccination	Diarrhea prior to death
RO15DJ	Shar Pei	F	3 months	Unvaccinated	Diarrhea prior to death
RO16DJ	French Bulldog	F	6 months	Unvaccinated	Diarrhea prior to death
RO17DJ	Puggle	F	10 months	Complete vaccination	Diarrhea prior to death
RO18DJ	Poodle	M	3 months	Complete vaccination	Diarrhea prior to death
RO19DJ	German Shepherd	M	3 months	Complete vaccination	Diarrhea prior to death
RO20DJ	Mixed breed	M	6 months	Unvaccinated	Diarrhea prior to death
RO21DJ	Mixed breed	F	10 months	Unvaccinated	Sudden death; previous clinical signs not documented
RO22DJ	Poodle	F	10 months	Complete vaccination	Diarrhea prior to death
RO23DJ	Mixed breed	F	3 months	Unvaccinated	Diarrhea prior to death
RO24DJ	Mixed breed	M	11 months	Incomplete vaccination	Sudden death; previous clinical signs not documented
RO25DJ	Golden Retriever	F	10 months	Unvaccinated	Diarrhea prior to death
RO26DJ	Mixed breed	F	10 months	Incomplete vaccination	Diarrhea prior to death

RO27DJ	German Shepherd	M	6 months	Complete vaccination	Diarrhea prior to death

Legend: Clinical information is reported according to the available submission forms and medical records. A history of diarrhea prior to death was documented in 22/27 dogs (81.5%). In 5/27 dogs (18.5%), the animals were submitted following sudden death, and previous clinical signs were not documented. The absence of documented clinical signs should not be interpreted as confirmation that the animals were clinically asymptomatic.

Table S2: Pathological findings and PCR-based detection of CPV-2 DNA in myocardial tissue samples from young dogs

Samples Identification	Heart lesions/Histological aspects	Other observations	**PCR results**
RO01DJ	Cardiomyocyte necrosis, myocarditis, and myocardial fibrosis. Heart pronounced persistent, scattered, and regional degeneration and replacement of myofibers with fibrous connective tissue. The cardiac ventricular wall showed pallor.	The inflammatory infiltrates are macrophages, lymphocytes, plasma cells, and neutrophils. Alveolar histiocytosis, liver centrilobular congestion, and hepatic lipid vacuolar alteration. There are no observable histologic alterations in the intestines.	positive
RO02DJ	Myocarditis, myocardial fibrosis	The inflammatory infiltrates are macrophages, lymphocytes, plasma cells, and neutrophils. Alveolar histiocytosis, liver centrilobular congestion. There are no observable histologic alterations in the intestines.	negative
RO03DJ	Myocarditis and myocardial fibrosis. Heart pronounced persistent, scattered, and regional degeneration and replacement of myofibers with fibrous connective tissue. The cardiac ventricular wall showed pallor.	The inflammatory infiltrates are macrophages, lymphocytes, plasma cells, and neutrophils. Alveolar histiocytosis, liver centrilobular congestion. There are no observable histologic alterations in the intestines.	positive
RO04DJ	Myocarditis	The inflammatory infiltrates are macrophages, lymphocytes, plasma cells, and neutrophils. Alveolar histiocytosis, liver centrilobular congestion. There are no observable histologic alterations in the intestines.	negative
RO05DJ	Cardiomyocyte necrosis, myocarditis, and myocardial fibrosis	The inflammatory infiltrates are macrophages, lymphocytes, plasma cells, and neutrophils. Alveolar histiocytosis, liver centrilobular congestion, and hepatic lipid vacuolar alteration. There are no observable histologic alterations in the intestines.	positive
RO06DJ	Myocarditis	The inflammatory infiltrates: macrophages, lymphocytes, plasma cells, and neutrophils	negative
RO07DJ	Cardiomyocyte necrosis, myocarditis, and myocardial fibrosis. Heart pronounced persistent, scattered, and regional degeneration and replacement of myofibers with fibrous connective	The inflammatory infiltrates are macrophages, lymphocytes, plasma cells, and neutrophils. Alveolar histiocytosis, liver centrilobular congestion, and hepatic lipid vacuolar alteration. There are no observable histologic alterations in the intestines.	positive

	tissue. The cardiac ventricular wall showed pallor.		
RO08DJ	Myocarditis	The inflammatory infiltrates are macrophages, lymphocytes, plasma cells, and neutrophils. Alveolar histiocytosis, liver centrilobular congestion. There are no observable histologic alterations in the intestines.	negative
RO09DJ	Myocarditis	The inflammatory infiltrates: macrophages, lymphocytes, plasma cells, and neutrophils	negative
RO10DJ	Cardiomyocyte necrosis, myocarditis, and myocardial fibrosis. Heart pronounced persistent, scattered, and regional degeneration and replacement of myofibers with fibrous connective tissue. The cardiac ventricular wall showed pallor.	The inflammatory infiltrates are macrophages, lymphocytes, plasma cells, and neutrophils. Alveolar histiocytosis, liver centrilobular congestion, and hepatic lipid vacuolar alteration. There are no observable histologic alterations in the intestines.	positive
RO11DJ	Myocarditis	The inflammatory infiltrates are macrophages, lymphocytes, plasma cells, and neutrophils. Alveolar histiocytosis, liver centrilobular congestion. There are no observable histologic alterations in the intestines.	negative
RO12DJ	Cardiomyocyte necrosis, myocarditis, and myocardial fibrosis	The inflammatory infiltrates are macrophages, lymphocytes, plasma cells, and neutrophils. Alveolar histiocytosis, liver centrilobular congestion, and hepatic lipid vacuolar alteration. There are no observable histologic alterations in the intestines.	positive
RO13DJ	Myocarditis	The inflammatory infiltrates: macrophages, lymphocytes, plasma cells, and neutrophils	negative
RO14DJ	myocarditis, and myocardial fibrosis	The inflammatory infiltrates are macrophages, lymphocytes, plasma cells, and neutrophils. Alveolar histiocytosis, liver centrilobular congestion, and hepatic lipid vacuolar alteration. There are no observable histologic alterations in the intestines.	positive
RO15DJ	Myocarditis	The inflammatory infiltrates are macrophages, lymphocytes, plasma cells, and neutrophils. Alveolar histiocytosis, liver centrilobular congestion. There are no observable histologic alterations in the intestines.	negative
RO16DJ	Myocarditis	The inflammatory infiltrates: macrophages, lymphocytes, plasma cells, and neutrophils	negative
RO17DJ	Cardiomyocyte necrosis, myocarditis, and myocardial fibrosis. Heart pronounced persistent, scattered, and regional degeneration and replacement of myofibers with fibrous connective tissue. The cardiac ventricular wall showed pallor.	The inflammatory infiltrates are macrophages, lymphocytes, plasma cells, and neutrophils. Alveolar histiocytosis, liver centrilobular congestion, and hepatic lipid vacuolar alteration. There are no observable histologic alterations in the intestines.	positive

RO18DJ	Myocarditis, myocardial fibrosis	The inflammatory infiltrates are macrophages, lymphocytes, plasma cells, and neutrophils. Alveolar histiocytosis, liver centrilobular congestion. There are no observable histologic alterations in the intestines.	negative
RO19DJ	Myocarditis	The inflammatory infiltrates are macrophages, lymphocytes, plasma cells, and neutrophils. Alveolar histiocytosis, liver centrilobular congestion. There are no observable histologic alterations in the intestines.	negative
RO20DJ	Myocarditis, myocardial fibrosis	The inflammatory infiltrates are macrophages, lymphocytes, plasma cells, and neutrophils. Alveolar histiocytosis, liver centrilobular congestion. There are no observable histologic alterations in the intestines.	negative
RO21DJ	Myocarditis	The inflammatory infiltrates are macrophages, lymphocytes, plasma cells, and neutrophils. Alveolar histiocytosis, liver centrilobular congestion. There are no observable histologic alterations in the intestines.	negative
RO22DJ	Myocarditis	The inflammatory infiltrates are macrophages, lymphocytes, plasma cells, and neutrophils. Alveolar histiocytosis, liver centrilobular congestion. There are no observable histologic alterations in the intestines.	negative
RO23DJ	Cardiomyocyte necrosis, myocarditis, and myocardial fibrosis	The inflammatory infiltrates are macrophages, lymphocytes, plasma cells, and neutrophils. Alveolar histiocytosis, liver centrilobular congestion. There are no observable histologic alterations in the intestines.	negative
RO24DJ	Myocarditis	The inflammatory infiltrates are macrophages, lymphocytes, plasma cells, and neutrophils. Alveolar histiocytosis, liver centrilobular congestion. There are no observable histologic alterations in the intestines.	negative
RO25DJ	Cardiomyocyte necrosis, myocarditis, and myocardial fibrosis. Heart pronounced persistent, scattered, and regional degeneration and replacement of myofibers with fibrous connective tissue. The cardiac ventricular wall showed pallor.	The inflammatory infiltrates are macrophages, lymphocytes, plasma cells, and neutrophils. Alveolar histiocytosis, liver centrilobular congestion, and hepatic lipid vacuolar alteration. There are no observable histologic alterations in the intestines.	positive
RO26DJ	Myocarditis	The inflammatory infiltrates are macrophages, lymphocytes, plasma cells, and neutrophils. Alveolar histiocytosis, liver centrilobular congestion. There are no	negative

		observable histologic alterations in the intestines.	
RO27DJ	Cardiomyocyte necrosis, myocarditis, and myocardial fibrosis	The inflammatory infiltrates are macrophages, lymphocytes, plasma cells, and neutrophils. Alveolar histiocytosis, liver centrilobular congestion. There are no observable histologic alterations in the intestines.	negative

Table S3: Distribution of myocardial histopathological lesions according to CPV-2 DNA detection status

Type of cardiac lesion	CPV-2 positive heart tissue samples (n = 9)	CPV-2 negative heart tissue samples (n = 18)	Total heart tissue samples (n = 27)
Cardiomyocyte necrosis	7 (77.78%)	2 (11.11%)	9 (33.33%)
Myocardial fibrosis	9 (100.00%)	5 (27.78%)	14 (51.85%)
Myocarditis	9 (100.00%)	18 (100.00%)	27 (100.00%)
Combination of cardiac lesions*	9 (100.00%)	5 (27.8%)	14 (51.9%)

*Combination of cardiac lesions was defined as the simultaneous presence of two or more histopathological lesions in the same myocardial sample, such as myocarditis with fibrosis or myocarditis with fibrosis and cardiomyocyte necrosis.

Legend: Histopathological lesions were recorded only when clearly identified in myocardial sections. Myocarditis was defined by the presence of inflammatory infiltrates associated with myocardial tissue damage. Degenerative, necrotic, inflammatory, and fibrotic changes were recorded separately to avoid overlapping lesion categories.

Figure S1: PCR detection of CPV-2 DNA in myocardial tissue samples (samples S1–S12).

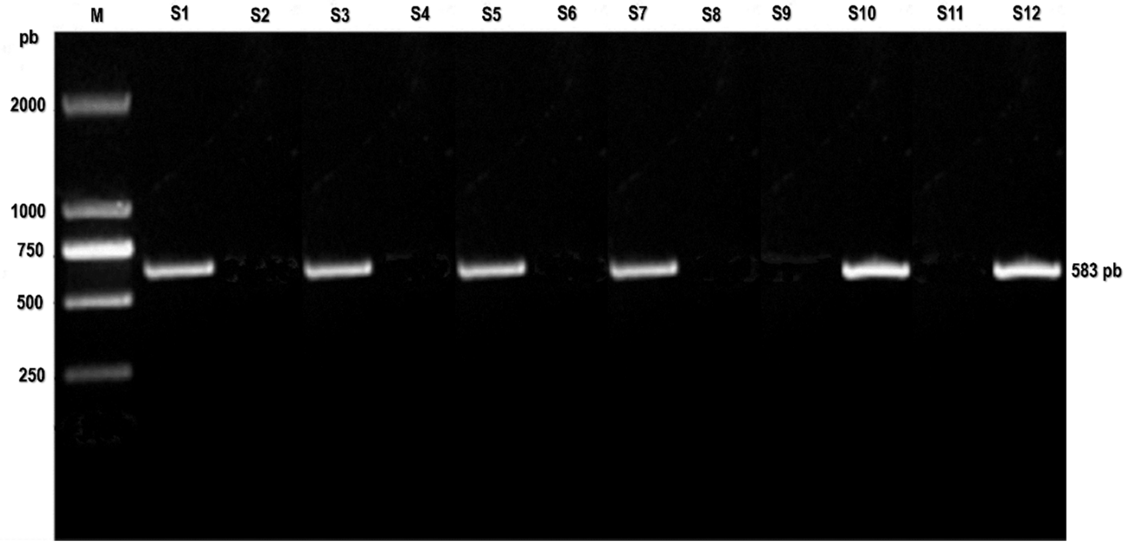

Legend: Agarose gel electrophoresis of PCR products amplified from FFPE cardiac tissue for CPV-2 DNA detection. M: DL2000 DNA ladder; PC: positive control; lanes S1–S12: myocardial tissue samples.

Figure S2: PCR detection of CPV-2 DNA in myocardial tissue samples (samples S13–S24).

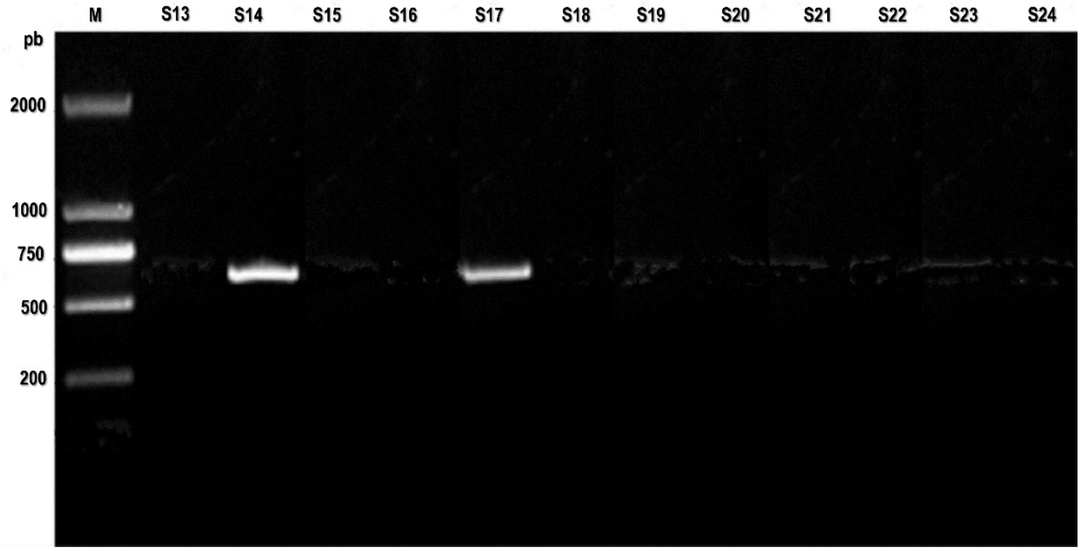

Legend: Agarose gel electrophoresis of PCR products amplified from FFPE cardiac tissue for CPV-2 DNA detection. M: DL2000 DNA ladder; lanes S13–S24: myocardial tissue samples.

Figure S3: PCR detection of CPV-2 DNA in myocardial tissue samples (samples S25–S27).

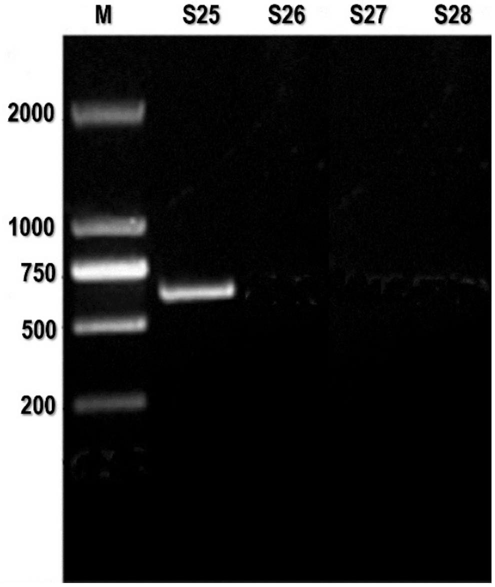

Legend: Agarose gel electrophoresis of PCR products amplified from FFPE cardiac tissue for CPV-2 DNA detection. M: DL2000 DNA ladder; lanes S25–S27: myocardial tissue samples; NC: negative control.
